# Application of nanoparticles in cancer detection by Raman scattering based techniques

**DOI:** 10.1080/20022727.2017.1373551

**Published:** 2017-12-19

**Authors:** Rouhallah Ravanshad, Ayoob Karimi Zadeh, Ali Mohammad Amani, Seyyed Mojtaba Mousavi, Seyyed Alireza Hashemi, Amir Savar Dashtaki, Esmail Mirzaei, Bijan Zare

**Affiliations:** a Department of Medical Nanotechnology, School of Advanced Medical Sciences and Technologies, Shiraz University of Medical Sciences, Shiraz, Iran; b Pharmaceutical Sciences Research Center, Shiraz University of Medical Sciences, Shiraz, Iran; c Department of Medical Biotechnology, School of Advanced Medical Sciences and Technologies, Shiraz University of Medical Sciences, Shiraz, Iran

**Keywords:** Nanoparticles, cancer detection, Raman technics, biomarkers

## Abstract

In vitro detection technique Raman spectroscopy (Rs), in one number times another Rs  based expert ways of art and so on, are useful instruments for cancer  discovery. top gave greater value to Raman spectroscopy  sers is a relatively new careful way for in vitro  and in vivo discovery that takes away bad points of simple Raman spectroscopy (Rs). Raman spectroscopy (RS) and in particular, multiple RS-based techniques are useful for cancer detection. Surface enhanced Raman spectroscopy (SERS) is a relatively new method for both *in vitro* and *in vivo* detection, which eliminates the drawbacks of simple RS. Using nanoparticles has elevated the sensitivity and specificity of SERS. SERS has the potential to increase sensitivity, specificity and spatial resolution in cancer detection, especially in cooperation with other diagnostic imaging tools such as magnetic resonance imaging (MRI) and PET-scan polyethylene terephthalate. Developing a hand held instrument for detecting cancer or other illnesses may also be feasible by using SERS. Frequently, novel nanoparticles are used in SERS. With a focus on nanoparticle utilization, we review the benefits of RS in cancer detection and related biomarkers. With a focus on nanoparticles utilizations, the benefits of RS in cancer detection and related biomarkers were reviewed. In addition, Raman applications to detect some of prevalent were discussed. Also more investigated cancers such as breast and colorectal cancer, multiple nanostructures and their possible special biomarkers, especially as SERS nano-tag have been reviewed. The main purpose of this article is introducing of most popular nanotechnological approaches in cancer detection by using Raman techniques. Moreover, have been caught up on detection and reviewed some of the most prevalent and also more investigated cancers such as breast, colorectal cancer, multiple intriguing nanostructures, especially as SERS nano-tag, special cancer biomarkers and related approaches. The main purpose of this article is to introduce the most popular nanotechnological approaches in cancer detection by using Raman techniques.

## 1. Introduction

Increasing promotions of medical science are promising for complete treatment of a violent group of ailment that human being struggle until now and known under a famous label, the cancer. In a minimalist look, these advancements will lead an abatement of patient's afflictions. Although cancers in physiology, motives, manifestations, prognosis, steps and severity of sign and symptoms, are very different, but in summary and a part of cancer type, abnormal proliferation and growth of involved organ or organ cells are the  main causes of it and would be established by invasion or spread to other part of the body. According to the GLOBOCAN database in 2012, there were an estimated 14.1 million new cases of cancer in the world[s8] , that lung, breast and bowel cancers (including anus), were most prevalent cancer cases. Early detection, with all its proper subsets, is essential for effective and perfect treatment of cancer [1,2]. Advances in medical science show promise in the treatment of cancer. Cancer varies in physiology, manifestation, prognosis, severity and symptoms. Regardless of the type of cancer, abnormal proliferation and cell growth of an organ or organs are two main causes, and these may be followed by the cancer spreading to other parts of body. According to the GLOBOCAN database, in 2012 there were about 14.1 million new cases of cancer in the world, among which lung, breast and bowel cancers (including anus) have been the most prevalent. Early detection of cancers is very important for treatment to help control the growth of malignant tissues [3].

Cancer detection with a handheld instrument is now possible [4]. Raman spectroscopy (RS) is a powerful analytical technique that can be used in the detection of biological specimens, even a single cell. Though RS is not a novel technology, its application for cancer detection is new. Recent advances in RS include *in vivo* trials [4–6]. RS can measure the chemical composition of complex biological samples [7]. Similar to wave-dependent imaging techniques, RS can provide real-time (or near real-time) molecular information and high resolution imaging at relatively low cost compared to other well-established medical imaging techniques (e.g. ultrasound and magnetic resonance imaging (MRI)) [8]. RS yields a spectrum containing several peaks, each of which is characteristic of a specific molecular bond. Collectively, these peaks provide an intrinsic ‘molecular fingerprint’ of the sample (the Raman spectrum), resulting in information about the chemical bonds associated with various molecules. Therefore, the advantages of Raman scattering based techniques include determining chemical specificity without the use of labels, being non-invasive, and having a very good spatial resolution . However, there are significant drawbacks related to the weak signal, a much stronger fluorescence background, and a limited penetration depth, which is typical for optical techniques. Several Raman based techniques have been developed to address these limitations and to perform measurements in different situations [9].

**Figure 1.**
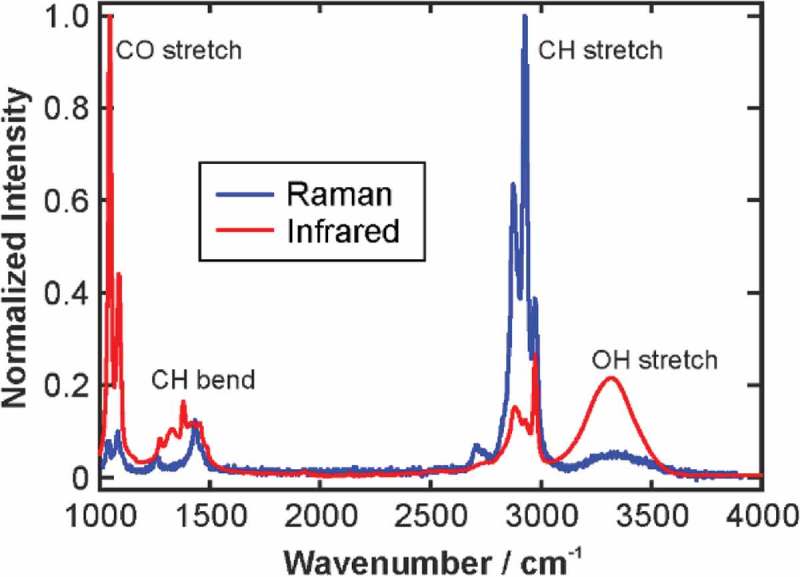
A typical Raman spectrum of a chemical compound and related peaks [10].

Surface enhanced Raman spectroscopy (SERS) is an advanced technique that in combination with nanotechnology enables highly accurate target detection. Silver and gold are the most popular nanoparticles which are used in SERS, different nanostructures, companionship with other nanoparticles, coating materials, biomolecules to aim various biomarkers and tumors (Figure 1) [10]. Nanostructures studied include Au-Ag nanoshells synthesised by galvanic exchange reaction of citrate-reduced Ag nanoparticles (NPs), as well as solid citrate-reduced Ag and Au NPs, both before and after stabilization with polymer . All nanostructures were characterized in terms of their size, surface plasmon resonance wavelength, surface charge, and chemical composition.The differential SERS activity of polymer-stabilized nanostructures is a result of discriminatory binding of analytes within-adsorbed polymer monolayer and a subsequent increase of analyte concentration at the nanostructure surface.

## 2. Principles of Raman spectroscopy

‘Raman scattering’ is a type of secondary radiation (the radiation emitted by molecules or atoms after bombardment by a primary radiation). When an incident photon hits a molecule, it can be absorbed or scattered. The photon may scatter in two ways: elastic and inelastic. If the scattered photon has the same energy as the incident photon, then this type of dissipation is called elastic; otherwise inelastic scattering occurs. Photons which experience inelastic collisions with molecules cause not only an exchange of energy, but also a change in frequency. Moreover, the difference of energy excites the molecules in the ground state. This brings the molecule into a virtual energy state for a short period before inelastic scattered photon fallout. The scattered photon may have lower or higher energy than the incoming photon. The scattered photons with lower energy are called Stokes and those with higher energy are called anti-Stokes. The absorbed energy causes rotational and vibrational changes. These changes lead to a change in the molecular dipole-electric polarizability. The intensity of the Raman scattering is proportional to dipole polarizability changes. This contrasting feature allows one to analyze transitions that might not be FTIR active, via RS. In Raman spectroscopy typically uses a non-ionizing laser as the excitation source. Selection of a suitable source needs to consider properties of the sample and spectrometer. As this process is generally weak, because of a very limited number of scattered photons (approximately one in every 10^6^–10^8^ scattered photons), it has a very small scattering cross section which is 12–14 orders of magnitude lower than that of fluorescence. Some optimizations were performed in this method. As a result, several variations of RS such as resonance Raman (RR), coherent anti-Stokes Raman scattering (CARS) and surface-enhanced Raman scattering (SERS) have been developed, to enhance the sensitivity and to take stronger signals.

RS is a flexible technique that allows a wide range of samples to be investigated, including solids, semi-solids, suspensions, and solutions, even through transparent, translucent, and opaque containers. Analysis of biological samples is enhanced with a minimum requirement for preparation and invasive manipulation. Aqueous solutions can easily be analyzed and water is not an obstacle in IR spectroscopy. Other advantages of this method are: very low concentrations (picomol) may be detected by optimized Raman instruments, Raman spectra are acquired quickly within seconds; laser light and Raman dissipated light can be dispatched by optical fibers over long distances for remote surveys. However, detection by this technique needs sensitive and highly optimized instrumentation, Raman spectra can be concealed by fluorescence of impurities of the specimens, laser radiation can heat and destroy the sample or cover the Raman spectrum, and as mentioned previously, spontaneous Raman signals are very weak. In spite of all of these disadvantages, the technique has been optimized, e.g. by selection of longer wavelength excitations for less fluorescence background productions or using noble metals in SERS to resolve weak signal problems. In addition, SERS optimization has been considered in the detection of biological materials [8,9,11–22].

**Figure 2.**
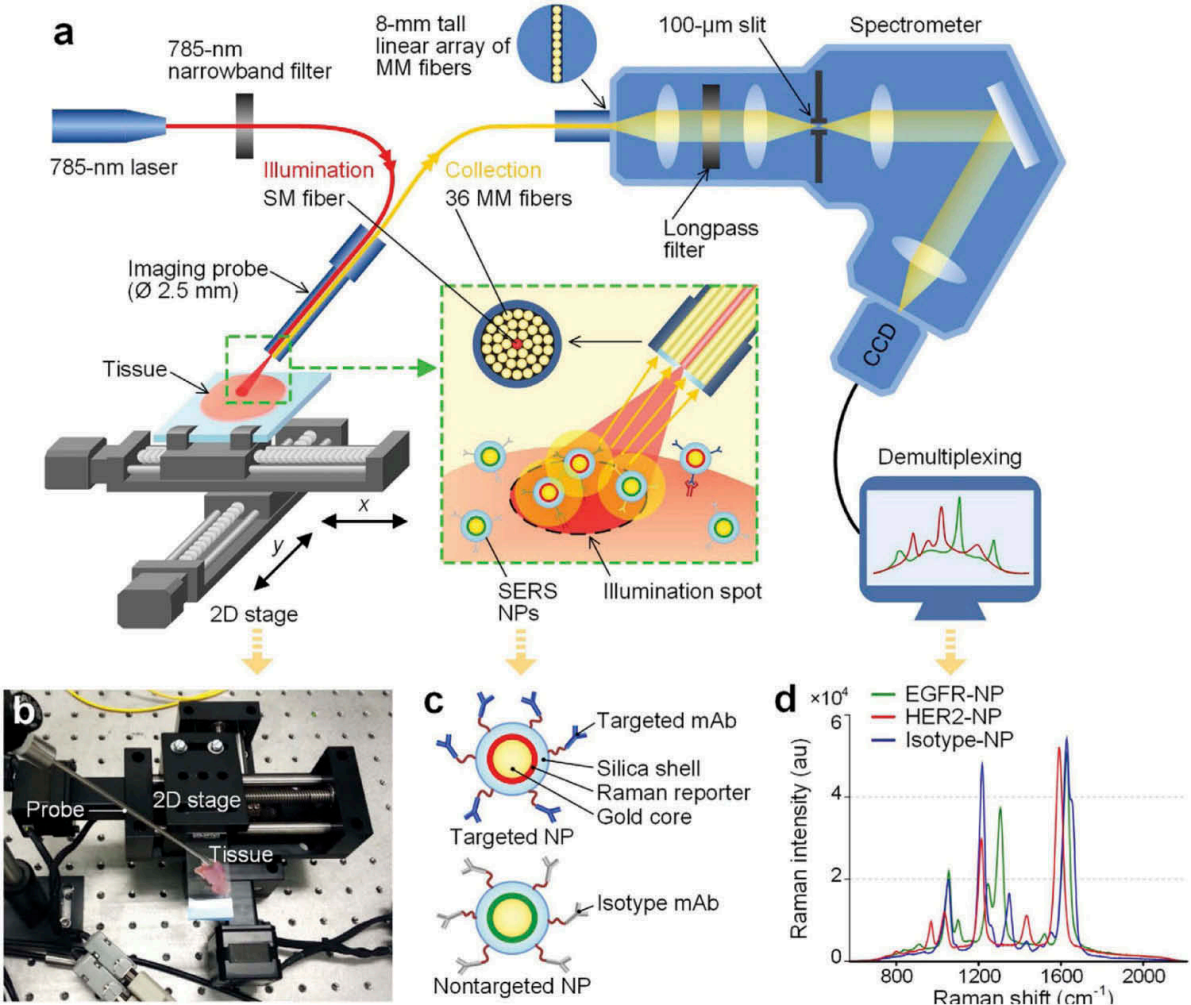
Schematic of the Raman spectra-imaging system. A 785 nm laser is used to illuminate the NP-stained tissue, creating a submillimeter-diameter laser spot. Raman-scattered photons from illuminated NPs are collected by multimode fibers and transmitted to a customized spectrometer (Andore Holospec®), where they are dispersed onto a cooled deep-depletion spectroscopic CCD. For raster scanning imaging, two axes are controlled through a custom LabVIEW program to translate the tissue sample. (b) A photograph of the raster-scanned tissue-imaging device. (c) A depiction of targeted SERS NPs and Biomarker-targeted surface-enhanced Raman scattering (SERS) nanoparticles (NPs) have been explored as a viable option for targeting and imaging multiple cell-surface  biomarkers of cancer. (d) The Raman spectra of the various SERS NPs used in a related study [31].

SERS is a plasmatic effect [1,23]; the term is derived from plasmon. When conductive electrons of metals (mostly noble metals), due to impaction of an electromagnetic beam (e.g. a laser beam), oscillate in a frequency equal to the impinging irradiation, the oscillating electrons are called ‘surface plasmons’. As long as metallic nanoparticles are used with a smaller size than the wavelength of the incident light, surface plasmons can be excited, leading to an increase in scattered light and electromagnetic field inside and near the particles [24–29]. When the target molecules are adsorbed into a roughened surface of nano-noble metals, a severe increase in the incident electromagnetic field and chemical charge transfers occurs, resulting in more intensive Raman signals (10^6^–10^14^ times over that of a flat surface) [17,30]. This allows the detection of organic coupled nanoparticles at picomolar concentrations even in deep tissues and makes it an ideal *in vivo* detector (Figure 2) [4,31].

New investigations have demonstrated that up to five spectral signatures can be recognized and spectrally separated simultaneously in living subjects. Hence, a specific signature that is associated with a specific targeting ligand of the molecular profile of the cancer (i.e. a peptide, monoclonal antibody, affibody, or aptamer) which is coupled to the nanoparticle, can be determined by spectrally separating the SERS signatures in the detected signals from a tumor [32]. The SERS technique has excellent selectivity, rapid detection capability, high signal-to-noise ratio, non-photo bleaching features, and the use of single photo-excitation [20]. Another way to obtain larger Raman scattering enhancements, which is in close relationship with SERS, is to put the target molecule in the fractal space between aggregated colloidal nanoparticles, known as ‘hot spots’ [33–36]. Additionally, we can exploit SERS not only for detection of known biomarkers, but also to detect novel and potential cancer biomarkers. However, a major weakness of this technique is the lack of reliable SERS substrates. Currently available substrates usually contain irregular active sites that suffer from strong spatial and temporal fluctuation in Raman intensity, and thus do not produce stable Raman output [29]. Still, there are a few sample pretreatment methods, such as Western blot SERS, fluorescein isothiocyanate-linked SERS, and ligand SERS, that can increase the sensitivity of SERS detection [22,37].

## 3. Nanoparticles and Raman techniques

RS does not require nanoparticles; however, the application of them in combination with RS has made this technique a reliable detection tool, especially in various fields of biology (Table1). Multiple nano-probes were used in various studies, but noble metal nanoparticles compose the main portion of this nano-probe to use for detection purposes as better surface plasmon resonance (SPR) property. Multiple nano-probes were used in various studies, but noble metal nanoparticles compose the main portion of this nano-probe to use  Multiple nano-probes were used in various studies, A compelling motivation in surface science is the development of nanoscale probes to seamlessly interrogate the molecular-level properties of material interfaces. This goal envisions probes which both engage and respond to the local material environment. To this end, scanning probe microscopytechniques have revolutionized our experimental capabilities for surface metrology. Atomic force microscopy (AFM) is one of the most successful scanning probe microscopy techniques and employs a cantilever-mounted tip to probe atomic details of a surface.

**Table 1.** Some common nanoparticle structures, targeted cancer markers, and Raman reporters, in cancer detection using SERS.

Cancer marker	Detected cancer	SERS nanoparticle	Structure in brief	Detection specificity agent	RAMAN marker
PSMA [38]	Prostate	AuNPs	28nm popcorn-shaped (GNPOP)	PSMA antibody and A9 RNA anti-PSMA aptamer	Rh6G
HER2 [39]	Breast	AuNPs	GNPOP-attached SWCNT	S6 aptamer	Free dye labeling
CEA [40]	Lung	AuNPs	Hollow gold nano-spheres	CEA antibody	4,4*′*-DP
CEA [41]	Lung, prostate and others	Fe_3_O_4_-AuNPs	Fe_3_O_4_ NPs is coated with AuNPs	CEA antibody	4-ATP
CEA, AFP, CA12 [42]	Lung, prostate and others	Si@(AgNPs/PEI)	Cross-linking of small AgNPs at the surface of silica particle	Anti-CEA, anti-AFP and anti-CA125	4-ABT
FRs [43]	Various cancer types	g-C_3_N_4_/Au@ AgNPs	PEI functionalized g-C3N4 nanosheets and anchor the Au@AgNPs	Folic Acid	Rh6G
CD19 antigen [44]	Leukemia	AuNPs	Pegylated gold NPs	CD19 antibody	MGITC
EGFR [45]	Various cancer types	AuNPs	Immobilized SERS nanotag (MGITC bioconjugated AuNPs) in the core of hollow photonic crystal fiber	EGFR antibody	MGITC
CD34, SCA-1 [32]	Lung cancer	M-SERS DOTs	18nm magnetite in the core and AgNPs and silica as coating material	CD34-antibody, SCA1-antibody	4-MT and BT
CEA Tf [46]	CEA over expressed cancer cells (most of cancer cells)	PAH-PSS-Dye-GNRs	PAH layer on PSS layer create a two layer polyelectrolyte coated Gold Nan-Rods and Raman reporter directly connecting onto the surface of GNRs	CEA-8 and TfR	FITC, DTDC, DTTC, O170

Abbreviations: PSMA, prostate-specific membrane antigen; SWCNT, single wall carbon nanotube; Rh6G, rhodamine 6G; AuNP, gold nanoparticle; GNPOP, gold nano-popcorn; CEA, carcinoemberyonic antigen; 4,4′-DP, 4,4′-dipyridyl; FRs, folate receptors; g-C_3_N_4_, graphite- phase carbon nitride; AFP, alfa fetoprotein; CA125, carbohydrate antigen; DNBA, 5,5-dithiobis(2-nitrobenzoic acid); 4ATP, 4-aminothiophenol; MGITC, malachite green; CD34, cell differentiate antigen; SCA-1, stem cell antigen; 4-MT, 4-methyl benzenthiol; BT, benzenthiol; PSS, poly(styrene sulfonate); PAH, poly(allylamin hydrochloride); FITC, fluorescent isothiocyanate; DTTC, 3,3ˊ-diethylthiatricarbocyanine; DTDC, 3,3′-diethylthiadicarbocyanine; O170, oxazine170 perchlorate; GNRs, gold nanorod; Tf, transferrin; TfR, transferring; TF, transfer factor.

**Figure 3.**
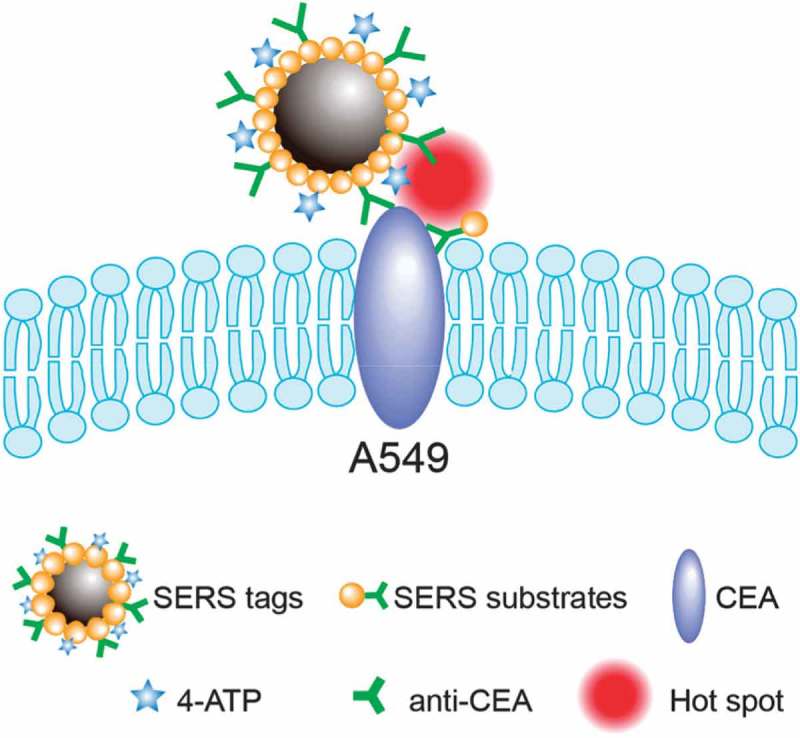
Schematic illustration of formation of sandwich structure and SERS HOT spot with both SERS tags (anti-CEA/4-ATP/Fe_3_O_4_-Au NPs) and SERS – active substrate [41].

SERS nano-probes (or nano-tags) usually have four major components: a noble metal (Au or Ag) nanostructure core for the SERS effect, Raman active dye molecules absorbed on the metal particle to contribute Raman signatures (SERS reporter), a surface coating layer necessary for the nano-probe’s stability and biocompatibility, and conjugated targeting ligands (e.g. antibodies) for selective binding of the nanoparticle to a specific target (Figure 3) [47]. SERS nanoparticles are less toxic, have narrower spectral peaks, and because of their unique spectral fingerprint, they can be easily resolved. Furthermore, their sensitivity *in vivo* is in the picomolar range [48]. Beqa et al. designed novel hybrid nanomaterials using a gold nano popcorn (GNPOP) -attached single-wall carbon nanotube (SWCNT) for targeted diagnosis and clearly demonstrated that S6 aptamer attached to the GNPOP-modified SWCNT-based SERS assay is highly selective for binding to SKBRk3 as human breast cancer cell line, which over express HER2. They compared this selective binding in MDA-MB as an HER2-negative breast cancer cell line. Due to the formation of aggregates in the presence of SKBR3 cells, it formed several hot spots, which provided significant enhancement of Raman signal intensity from SWCNTs by three orders of magnitude through electromagnetic field enhancements [39,44]. SERS nano-tags with metallic NPs in the form of nano rods, hollow nanostructures, and nano stars because of having an active hot spot could give out higher sensitivity that is useful for *in vivo* applications. SERS nano-tags also have many significant advantages over fluorescence based NPs like quantum dots, e.g. (i) multiplex detection capability due to spectral fingerprinting; (ii) not being vulnerable to photo-bleaching; and (iii) low cytotoxicity due to the usage of AuNPs [1].

Gold nanoparticles (AuNPs) have been used in imaging for more than three decades. Advantages of AuNPs when used in SERS includes SPR, size dependent specifications, easy surface modifications, lower toxicity associated with living cells, size and shape diversity. AuNPs can easily be functionalized with antibodies and other tumor targeting biomolecules through well-established conjugation procedures [49,50]. In recent years, several groups [51, 52] have demonstrated the potential of using AuNPs based on Raman tags for SERS. Depending on the location of the tumor tissue, a variety of AuNPs were  used for SERS. Count on the location of the tumor tissue, a variety of AuNPs were used for SERS. AuNPs with different sizes, shapes and optical properties were used for detection [53,54]. AuNPs can reach malignant tissues in two ways. The first is through a passive effect called enhanced permeability and retention (EPR): AuNPs accumulate in malignant tissues by permeating via leaky tumor vessels during circulation. The second is AuNPs attaching to targeted ligands (e.g. proteins, antibodies, aptamers, and small molecules), aiming for specific receptors, proteins and cell types [28,41,55,56]. Regarding the shape of AuNPs, some advantages were demonstrated when using star structures, but much work has also been done with spheres [4]. Silver nanoparticles (AgNPs) can also be used in correlation with RS and SERS. Although colloidal AuNPs are more stable than Ag colloids, the Raman signal enhancement effect of silver is 100–1000 times greater than that of gold, in addition to lower Ag cost [57]. However, the two nano-noble metals have not been greatly exploited, as can be seen by a search of relevant databases. Like for AuNPs, the structure of AgNPs and the modality of their interactions with analytes and each other is important. Takei et al [58]. concluded that existence of minor space between nanoparticles, a sufficient thickness of the silver nano-layer, and mixing of nanoparticle (spherical shape) with various diameters are important features to boost SERS presentation.

Some other nanostructures have been used for particular purposes. For instance, nanotubes can be used for cancer detection. Ingle et al. used functionalized single-walled carbon nanotubes (SWCNT) in the form of an aerosol in the lungs of mice to detect lung cancer [59]. Analysis showed that after the arrival of the aerosol to the lungs of mice, blood samples can be used for the detection based on SERS. In a hybrid method Fe_3_O_4_–Au nanoparticles were attached to 4-aminothiophenol (4-ATP) as SERS reporter and antibody of CEA (anti-CEA) to create (anti-CEA/4-ATP/Fe_3_O_4_-Au). A SERS reporter was used in conjunction with anti-CEA labeled AuNPs (anti-CEA/Au) as SERS active substrate, to form hot spots on the surface of CEA positive-expressed cancer cells (e.g. A549), to improve detection sensitivity [41].

## 4. Cancer detection using Raman

Early detection is essential for effective treatment of cancer. To meet this goal, screening tests play an important role. Specific screening tests have been developed for different cancer types, e.g. pap smear test for cervical carcinoma, mammography for breast cancer, and prostate-specific antigen (PSA) test test and rectal exam for prostate malignancies [60]. Furthermore, powerful techniques have been developed for *in vivo* cancer detection, e.g. computer tomography (CT)-scan, PET-scan (positron emission tomography), and MRI. Detection of malignant tissues and determination of the cancer stage with these tools can be confirmed or corrected with pathological assessments of excisional biopsies through surgical methods. Another aspect of cancer detection relates to histology, cytology and pathological techniques. It is obvious that detection is a continuous process and may be done before, during and after every step of treatment. Despite the efficiency of imaging methods in cancer diagnosis, there are some limitations such as insufficient sensitivity, specificity, spatial resolution, inability to detect small volume tumor extensions and lymph node metastasis, and precise determination of the cancer stage. Histopathological approaches are very time consuming, need biopsy, and may be costly. Hence, scientists attempt to optimize detection methods and create novel tools [55,61–68].

Whereas RS *in vivo* cancer detection has been exploited, Raman spectroscopy although is not a novel technology but its application for cancer detection is a new scope  and recent advances in RS have promoted it to a level at which in vivo trials are beginning to emerge [12–14] RS application as a histopathologic tool has been considered mostly now that it is often exploited for examination of biopsied tissues since they may contain very small amounts of material to be searched. Accordingly, among the numerous techniques based on Raman, SERS seems to be more capable of achieving this purpose [4,69].

## 5. Breast cancer

**Figure 4.**
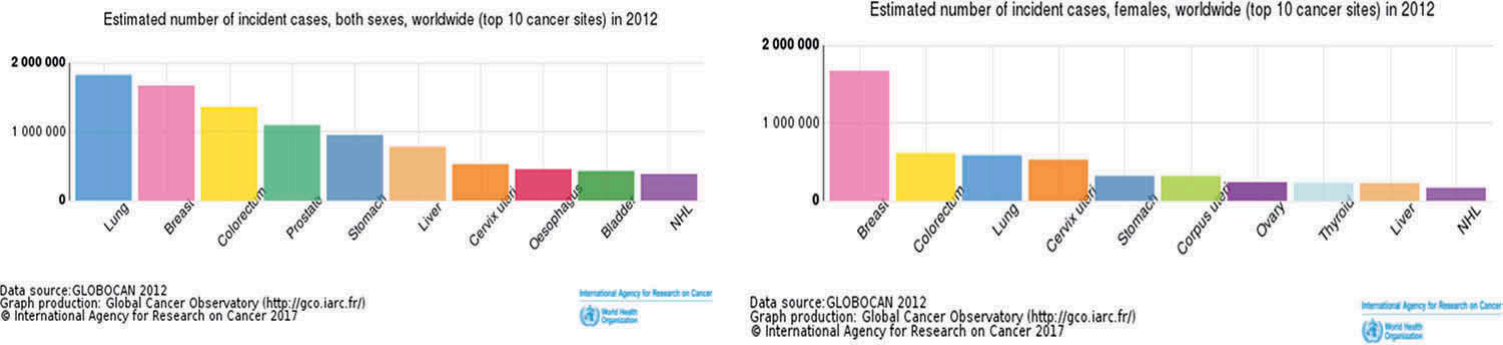
Graph of estimated number of different cancer cases. Breast cancer is by far the highest incident type among females [3].

Breast cancer presents the greatest challenge in today’s world health care. Globally, breast cancer is the most common cancer in women and the second most prevalent cancer in general after lung cancer. It has been estimated that in five years it will be the most common cancer (Figure 4) [3].

Several investigations have used RS to detect normal, precancerous and cancerous breast tissues. Most studies were done on breast cancer detection focusing on EGFR (epidermal growth factor receptor). Overexpression or amplification of human epidermal growth factor receptor 2 (HER2, a member of the EGFR family), also known as a tyrosine kinase transmembrane receptor, is a prognostic marker in many types of cancers. In breast cancer, a HER2-positive status is generally associated with a poor prognosis and a higher rate of disease recurrence compared with HER2-negative status. HER2 is an important biomarker, which plays an essential role in therapeutic decision making for breast cancer patients. Thus, identifying the HER2 status of breast cancer cells is very important for breast cancer therapies. Currently, the most commonly used methods for assessing HER2 status are immunohistochemistry and fluorescence *in situ* hybridization. Both methods suffer from several drawbacks such as being relatively time-consuming and not being suitable for direct *in vivo* detection. In this respect and to reduce the weakness, [70] used *p*-mercaptobenzoic acid (pMBA) molecules as both the Raman reporter (agents provide much higher Raman intensity) for generating SERS signals, and the conjugation agent for attaching antibody molecules to AgNPs. The method of nanoparticle preparation is straightforward because no shell is needed. The results showed that SKBR3 (as HER2-positive cells) exhibits much stronger SERS signals than MCF7 (as HER2 negative cells), indicating that the SERS probe potentially can distinguish the HER2 status of different kinds of breast cancer cells [69,71].

**Figure 5.**
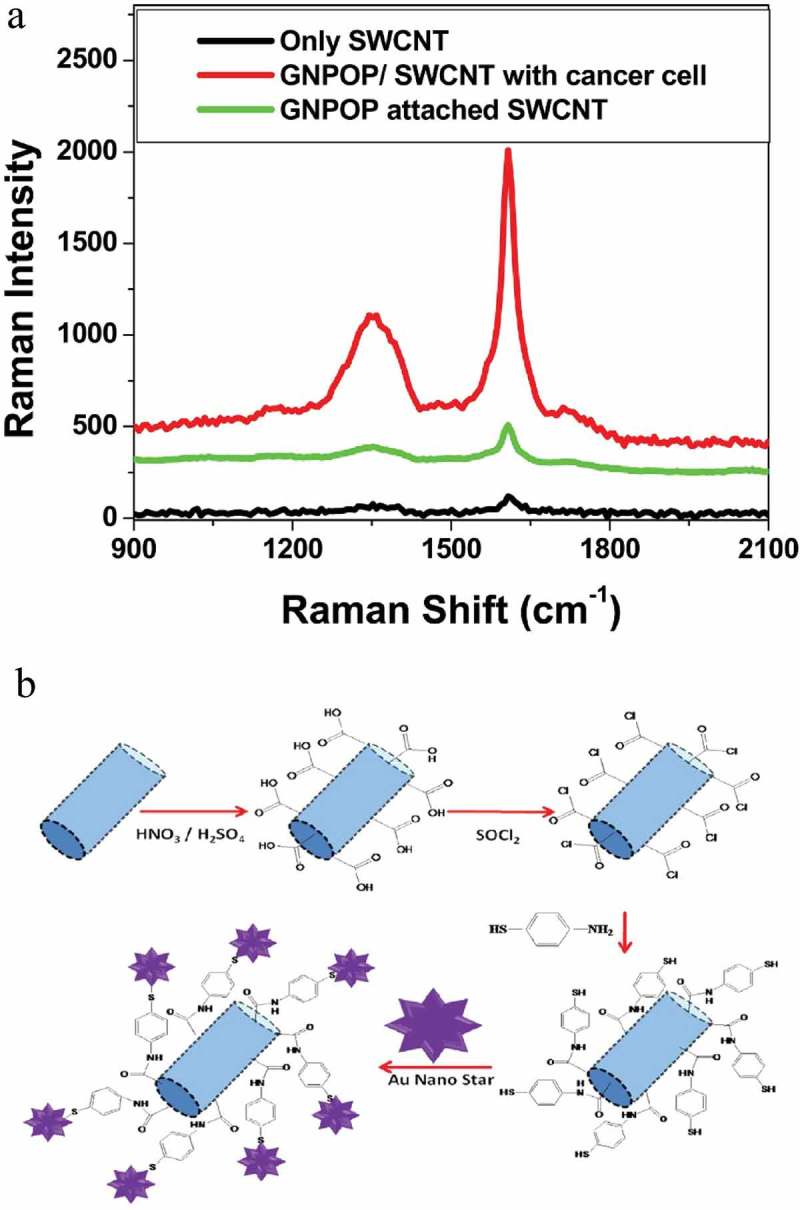
(a) Plot showing SERS. (b) Schematic representation shows the synthesis protocol for the formation of GNPOP attached SWCNTs [39].

**Figure 6.**
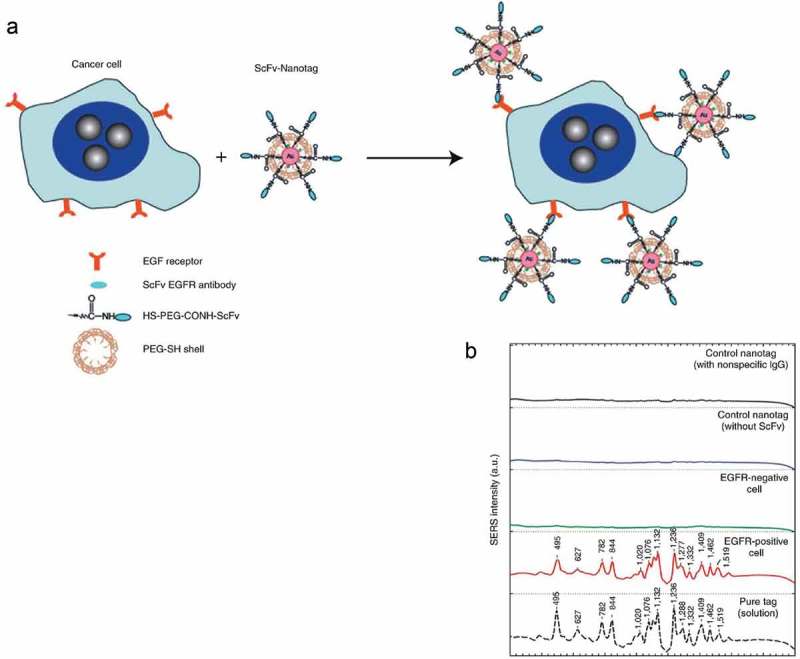
Cancer cell targeting and spectroscopic detection by using antibody-conjugated SERS nanoparticles. (a) Preparation of targeted SERS nanoparticles by using a mixture of SH-PEG and a hetero-functional PEG (SH-PEG-COOH). Covalent conjugation of an EGFR-antibody fragment occurs at the exposed terminal of the hetero-functional PEG. (b) SERS spectra obtained from EGFR-positive cancer cells (Tu686) and from EGFR negative cancer cells (human non-small cell lung carcinoma NCI-H520) together with control data and the standard tag spectrum. All spectra were taken in cell suspension with 785-nm laser excitation and were corrected by subtracting the spectra of nanotag-stained cells by the spectra of unprocessed cells. The Raman reporter molecule is diethylthiatri-carbocyanine (DTTC), and its distinct spectral signatures are indicated by wave numbers (cm^–1^) [71].

Other investigations use this format of detection with a focus on EGF ligand–receptor interaction. For example, [72,73] demonstrated a SERS probe for detecting circulating breast tumor cells using thiolate polyethylene glycol to improve the stability of the probe, and thiolate polyethylene glycol acetate to connect EGF to Au nanoparticle in order to distinguish breast cancer cells at different HER2 status [74]. In fact, the thiol-PEG-coated gold particles become so stable that their SERS signals do not change under very harsh conditions and, in comparison to near-infrared-emitting PEGylated SERS, nanoparticles were 4200 times brighter (on a particle-to-particle basis) under identical experimental conditions. Qian et al., in the first study of whole-animal Raman imaging in a multiplexed manner, exploited PEGylated Au nanoparticle and SERS, but they conjugated SERS nanoparticles with a ScFv antibody, a ligand that binds to EGFR with high specificity and affinity, for *in vitro* and *in vivo* tumor targeting. They asserted that their technique could detect deep tumors as accurately as superficial ones, and highlighted the sensitivity, specificity and non-toxicity of the technique and respective nanoparticles [51]. Dinish et al. [73] performed actively targeted multiplex in vitro and in vivo detection of three intrinsic cancer biomarkers, EGFR, CD44 and TGF beta-receptor II (TGFbRII), in a breast cancer model using three multiplexing capable and biocompatible SERS. antibody conjugated nanotags. Intra-tumorally injected antibody conjugated nanotags, specifically targeting the three biomarkers, exhibited maximum SERS signal at 6 h. These three SERS nano tags (SERS reporters) consist of cyanine 5 (Cy5) for (TGFbRII), malachite green isothiocyanate (MGIT) for CD44 and rhodamine6G (Rh6G) for EGFR, which are attached to gold nanoparticles (AuNPs) that were conjugated (with the mediation of polyethylene glycol (PEG)) to antibodies against these biomarkers. In their teranostic system, Zhao et al. innovated a SERS-active gold nano chain that had a suitable structure for potential application in multiplex detection and photodynamic therapy (PDT) of cancer [75]. They assembled Raman reporter (2-naphthalenethiol (NPT)) and Pheo-SH attached AuNPs with a biocompatible polymer conjugate hyaluronic acid–hydrocaffeic acid (HA–HCA) and made a chain-like structure. Multiplex detections could be exploited to enhance the specificity of cancer detection if we target multiple specific biomarkers of a tumoral tissue [38]. Zheng et al. evaluated the capability of SERS to identify different types of breast cancer from different sources. They used a previously developed technique, Au@sio_2_ shell-isolated nanoparticle-enhanced RS (SHINERS), to further distinguish between normal tissue and pathological states of fibroadenoma (FD), atypical ductal hyperplasia (ADH), ductal carcinoma *in situ* (DCIS), and invasive ductal carcinoma (IDC), in a comparative manner with special remarks on β-carotene and DNA spectra. They demonstrated that this technique could successfully characterize breast tissues and accurately distinguish five types of aforesaid breast cancer [76]. Other specifications for identifying malignant breast tissues include the study of Allain and Vo-Dinh, who exploited SERS to monitor DNA hybridization of a fragment of BRCA1 breast cancer susceptibility gene on modified silver surfaces that can be used in screening tests [77,78]. In another study, by using a SERS platform based on island film lithography combined with silver deposition by galvanic exchange and by using small, specific oligonucleotide sequences, Pang et al. developed a SERS gene probe for breast cancer [79]. Beqa et al. designed a novel hybrid nanomaterial using gold nano popcorn (GNPOP)-attached single wall carbon nanotube (SWCNT) for targeted diagnosis and clearly showed that S6 aptamer attached GNPOP-modified SWCNT based SERS assay is highly selective for binding with SKBR3 as a human breast cancer cell line, which overexpresses HER2. They compared this selective binding in MDA-MB as HER2-negative breast cancer cell line. Due to the formation of aggregates in the presence of SKBR3 cells, it formed several hot spots, which provided a significant enhancement of the Raman signal intensity from SWCNTs by three orders of magnitude through electromagnetic field enhancements (Figure 5) [39]. Other criteria can be used to detect breast cancer, In one paper, Matousek and Stone [80] showed the ability of Raman system to detect a calcified inclusion within a thick layer of tissue, with potential applications of breast cancer diagnosis (Figure 6).

## 6. Colorectal cancer

Colorectal cancer is the most prevalent and incidental malignancy after lung and breast cancer. It is a heavy financial burden on public healthcare systems in both developed and developing countries, especially because of aging and population growth. Approximately 50% of colorectal carcinomas arise in the rectum and recto sigmoid regions, 15% arises in the caecum and lower ascending colon, and the remainder are distributed roughly equally throughout the rest of the colon [4]. Like other malignancies, early diagnosis is essential for effective treatment. For this reason, screening methods have been developed to reduce the incidence by removal of adenomas, which can lead to a mortality rate reduction. Colonoscopy as a common screening method is very time consuming and needs a skilled examiner. During colonoscopy, polypoid lesions are commonly found. Adenomatous polyps are known to be pre-malignant and should prompt immediate removal. Despite many drawbacks, excisional biopsies currently remain the standard approach for cancer diagnosis. Excisional tissue biopsy carries risks of bleeding and visceral perforation as well as unnecessary removal of a normal tissue. Some disadvantages in conventional colorectal screening methods consider the necessity to develop new diagnostic methods. RS has been used for detection of colorectal cancer. RS is suitable for use with fiber-optic probes. It is potentially ideal as a medical diagnostic tool for assessment of hollow organs [4,81]. However, in early stages of colorectal cancer the epithelium of the luminal surface contain diagnostic  features while underlying tissues make fluorescence. To reduce the fluorescence background from the bulk tissues, Zheng et al. [82] used laser tweezers to trap a single cell from the epithelium and characterized it by using RS. Lin et al. [15] exploited the SERS technique by using gold colloid that was added to the sample serum of the recognized colorectal cancer cases and healthy cases (as a control group). They showed that the intensity of the many dominant vibrational bands increased, indicating a strong interaction between the gold colloid and the serum, significant Raman spectral differences, and also between normal and cancerous serums [15]. In another study, Campos da Paz et al. synthesized maghemite (Fe_2_O_3_, γ-Fe_2_O_3_) nanoparticles precoated with dimercaptosuccinic acid, and the surface, which functionalized with anticarcinoembryonic antigen (anti-CEA), successfully used to target those cell lines expressing characteristic CEA of colorectal cancer (CRC) cells. Then, surface enhanced RS (SERS) was used to track the surface decoration of the nanosized maghemite particles from the very first precoating up to the attachment of the anti-CEA moiety [83].

## 7. Brain cancer

RS could be used to detect brain cancer more accurately. CT-scan, MRI and electroencephalogram (EEG) are the most common brain tumor detection methods, and their findings are confirmed by surgical excision of suspicious tissues. About 45% of brain tumors are glioblastomas. Characterization of brain tissue is a priority to distinguish between malignant and healthy tissue, whether to trace the trend of progression of malignancy, for example in response to treatment, or to delineate brain tumor margin pre and intraoperatively for the precise resection of tumor [5]. In recent investigations, Koljenovic et al. [84] used a fiber-optic Raman probe with high wave number of laser beams (2400–3800 cm^−1^) to characterize brain tissue of a pig *ex vivo*. Raman spectra of the gray matter were characterized by high intensity band related to nucleic acids, proteins and phosphatidyl choline while the spectra of the white matter were associated with cholesterol, galactocerebroside and sphingomyeline [14]. Aydin et al. demonstrated the usefulness of SERS to differentiate healthy brain tissues from malignant ones. They directly mixed AgNPs with liquefied brain tissue, which was obtained from three different locations of the sample: the tumor, peripheral of the tumor and the healthy site of tissue samples. The dominant differences were seen of the ratio peaks at around 723cm^−1^ and 655cm^−1^ (I_723_/I_655_) [85, 86].

Constraints of current imaging techniques such as poor sensitivity, specificity and spatial resolution, necessitate novel methods that are able to resolve these limitations. In response to this demand, Kircher et al. applied innovative triple modality MRI, photoacoustic imaging, and Raman imaging nanoparticle (MPR nanoparticle) which can precisely depict the interface of brain tumors in living mice [87]. A combination of tumor localization, the macroscopic delineation ability of MRI, high spatial resolution, three-dimensional photoacoustic imaging, high sensitivity, high specificity and the high resolution surface imaging potential of RS produces a powerful and accurate method for delineating tumor margins. Aydin et al. designed a 60-nm gold core, covered with the Raman molecular tag trans-1,2-bis(4-pyridyl)-ethylene. The Raman-active outer layer is protected with a 30 nm silica coating. The particles then were modified with 1,4,7,10-tetraazacyclododecane-1,4,7,10-tetraacetic acid in complex with Gd^3+^ ((DOTA)-Gd^3+^) using a maleimide linkage (maleimide-DOTA-Gd). By using this method, the authors subsequently demonstrated not only its ability to yield high resolution brain tumor imaging and visualization of the margins of an invasive tumor, but also its use intraoperatively for brain tumor resection and to check unremoved malignant remainders as a probe [85,87]. Diaz and his coworker applied transcranial focused ultrasound to achieve maximal surgical resection and facilitate passing of a multifunctional designed gold nanoparticle (this was created by them to label and track glioblastoma cells through blood brain barrier (BBB) which serves as a barrier to nanoparticle transition from the vascular lumen to the brain parenchyma). This kind of ultrasound is shown to disrupt the BBB in a focal and reversible manner, and its potential application to brain tumor therapy has been recently demonstrated in rat models. They used MRI-guided transcranial focused ultrasound to demonstrate that PEG coated GNPs, which used for SERS imaging, can be delivered through the BBB [88].

## 8. Detection of other malignancies

Characterization of brain tissue is a priority for distinction between malignant and healthy tissue whether, for tracing  the trend of progression of malignancy for example in response to treatment or to delineate brain tumor margin pre and intraopratively in order to precise resection of tumor [13]. In previous sections cancer detection was reviewed by focusing on RS and nanoparticles and according to common malignancies, general principles of other cancer detection by this approach are nearly the same. In this section we briefly discuss the usages of this method and its capability.

Gastric cancer is one of the other malignancies that were explored by SERS technique. Feng et al. [89] exploited different polarity laser light and demonstrated that left-handed circularly polarized laser excitation has great promise of becoming a clinically useful diagnostic tool for non-invasive gastric cancer detection. Tang et al. [90] introduced gold nanoparticles into live human osteosarcoma cells by endocytosis and adding them to the growth medium. By using SERS cell mapping, they suggested the feasibility of the intracellular distribution of chemotherapeutic agent measurement. Qiu et al. successfully differentiated living normal and cancer cells by SERS imaging. They used a functional double-probe based on Ag/Au core/shell NPs. Rhodamine6G was used as SERS reporter and a monoclonal antibody specific to PLCγ1 marker that is over expressed in HEK293 cancer cells. PLCγ1 in competition with GRB2 to attach to FGFR2, that provides a binding site for these two proteins, and determines prognosis of recurrence-free survival in lung adenocarcinoma [19,33,41]. Bei et al.explored AgNP-based, SERS applications for biochemical analysis of blood plasma samples and demonstrated the capability of this method to detect nasopharyngeal cancer [91].

By using principal component analysis (PCA) combined with linear discriminate analysis (LDA) that was performed on the measured blood plasma SERS spectra, they showed the ability of this technique to produce high sensitivity (90.7%) and specificity (100%) [91]. The results suggested a great potential for SERS in nasopharyngeal cancer screening application [92]. Pang et al. used Fe_3_O_4_@Ag magnetic nanoparticle as microRNA (miRNA) capture to create an ultrasensitive detection tool in total RNA extraction from cancer cells in both SERS and duplex-specific nuclease signal amplification platform. Fe_3_O_4_@Ag serves both as SERS and as a concentration device [93]. Tianxi Yang et al. [94] used Fe_3_O_4_/Au/Ag nanocomposites-based SERS to detect adenosine (as a possible biomarker for malignancies) in urine from a patient with lung cancer using a portable Raman spectrometer. Tracking of a specific biomarker, as already seen, is another popular method in cancer detection. A growing number of researches pursue detection of mutant p53. This plays a crucial role in tumor suppression in the prevention of genome mutations. Thus, mutation of its genome may increase the risk of malignancy outbreak, so procedures for detecting very low concentration of this protein have been considered. The traditional approach to detect proteins including p53, i.e. enzyme-linked immunosorbent assay (ELIZA), in spite of acceptable accuracy, is very time-consuming. Dominique and his coworker used SERS technique in an analytical framework to develop a fast, simple, non-invasive and yet sensitive method for detection of mutant and wild-type p53. They used gold nanoparticle attached to 4-aminothiophenol (4-ATP) as SERS reporter [45,95,96]. Others exploited a similar approach for detection of malignant tissues or biomarkers. For example, Carvalho et al. [97] demonstrated the ability of Raman micro-spectroscopy to develop a rapid and non-invasive screening method of oral squamous cell carcinoma.

## 9. Conclusion

RS, and SERS in particular, is a powerful tool that can be used for detecting and tracing biological targets which are cell and biochemical compounds. Raman can be exploited in various experiments, not only as a probe instrument in the laboratory, but also for histological and pathological diagnosis in clinical affairs. And by designing various naostructures we can elevate sensitivity and specificity of detection, which is supremacy of it from other spectroscopic and even imaging techniques such as CT-Scan and MRI, exclusively in in-vivo biostructural detections. Raman based techniques are not yet proposed as routine instruments for diagnostic purposes even in clinical applications, probably due to the unfamiliarity of clinical laboratory specialists with the capabilities of these methods. More investigations are needed to adapt SERS and other Raman based techniques for clinical use.
